# NS3 from Hepatitis C Virus Strain JFH-1 Is an Unusually Robust Helicase That Is Primed To Bind and Unwind Viral RNA

**DOI:** 10.1128/JVI.01253-17

**Published:** 2017-12-14

**Authors:** Ting Zhou, Xiaoming Ren, Rebecca L. Adams, Anna Marie Pyle

**Affiliations:** a Department of Molecular, Cellular, and Developmental Biology, Yale University, New Haven, Connecticut, USA; b Department of Chemistry, Howard Hughes Medical Institute, Yale University, New Haven, Connecticut, USA; University of Southern California

**Keywords:** viral replication, evolution, hepatitis C virus, crystal structure, enzymology

## Abstract

Hepatitis C viruses (HCV) encode a helicase enzyme that is essential for viral replication and assembly (nonstructural protein 3 [NS3]). This helicase has become the focus of extensive basic research on the general helicase mechanism, and it is also of interest as a novel drug target. Despite the importance of this protein, mechanistic work on NS3 has been conducted almost exclusively on variants from HCV genotype 1. Our understanding of NS3 from the highly active HCV strains that are used to study HCV genetics and mechanism in cell culture (such as JFH-1) is lacking. We therefore set out to determine whether NS3 from the replicatively efficient genotype 2a strain JFH-1 displays novel functional or structural properties. Using biochemical assays for RNA binding and duplex unwinding, we show that JFH-1 NS3 binds RNA much more rapidly than the previously studied NS3 variants from genotype 1b. Unlike NS3 variants from other genotypes, JFH-1 NS3 binds RNA with high affinity in a functionally active form that is capable of immediately unwinding RNA duplexes without undergoing rate-limiting conformational changes that precede activation. Unlike other superfamily 2 (SF2) helicases, JFH-1 NS3 does not require long 3′ overhangs, and it unwinds duplexes that are flanked by only a few nucleotides, as in the folded HCV genome. To understand the physical basis for this, we solved the crystal structure of JFH-1 NS3, revealing a novel conformation that contains an open, positively charged RNA binding cleft that is primed for productive interaction with RNA targets, potentially explaining robust replication by HCV JFH-1.

**IMPORTANCE** Genotypes of HCV are as divergent as different types of flavivirus, and yet mechanistic features of HCV variants are presumed to be held in common. One of the most well-studied components of the HCV replication complex is a helicase known as nonstructural protein 3 (NS3). We set out to determine whether this important mechanical component possesses biochemical and structural properties that differ between common strains such as those of genotype 1b and a strain of HCV that replicates with exceptional efficiency (JFH-1, classified as genotype 2a). Indeed, unlike the inefficient genotype 1b NS3, which has been well studied, JFH-1 NS3 is a superhelicase with strong RNA affinity and high unwinding efficiency on a broad range of targets. Crystallographic analysis reveals architectural features that promote enhanced biochemical activity of JFH-1 NS3. These findings show that even within a single family of viruses, drift in sequence can result in the acquisition of radically new functional properties that enhance viral fitness.

## INTRODUCTION

Replication by hepatitis C virus (HCV) is carried out by a set of viral nonstructural (NS) proteins that include the replicative polymerase NS5B and a helicase enzyme, NS3 (1). The importance of NS3 in viral replication and packaging is well established (2), and the enzyme remains an important focus for the development of targeted therapeutics (3–6). In addition to its medical relevance, the NS3 helicase is among the most well-studied unwinding enzymes, and it has long been used as a model system for studying the molecular mechanism of helicase enzymes (7–9).

NS3 contains the typical structural features of a superfamily 2 (SF2) helicase, including the conserved sequence motifs for ATP hydrolysis and RNA recognition (7). These are arranged within two paradigmatic helicase domains, each of which adopts the RecA fold architecture that is typical for SF2 helicases. Two additional domains contribute to NS3 function, i.e., domain 3, which contains additional motifs for RNA binding and unwinding, and an N-terminally appended protease domain, which enhances helicase activity via a large, positively charged patch that contributes to high-affinity RNA binding (10). NS3 is an intriguing example of a dual enzyme in which the two components (helicase and protease) have evolved to become functionally interdependent (6, 10–13). Biochemical and biophysical studies with HCV genotype 1a (gt1a) and 1b helicases have shown that these enzymes unwind RNA duplexes that are adjacent to long, single-stranded 3′ overhangs (3′-tailed duplexes), which serve as a loading site for multiple NS3 molecules prior to ATP-dependent translocation and strand displacement (14, 15). These results led to the model that efficient RNA unwinding requires at least two NS3 molecules and a minimal 3′-overhang length of ∼18 nucleotides (nt) (15–20), although these NS3 monomers have a low level of intrinsic activity, particularly when the RNA duplex is under strain (16, 17). There is a pronounced lag in unwinding by the genotype 1a and 1b NS3 enzymes, which must undergo rate-limiting conformational changes in order to form a functional unwinding complex (5, 21). This behavior, along with the requirement for multimerization (15, 19, 20), significantly reduces the apparent activity of the genotype 1a and 1b NS3 enzymes and led to the notion that NS3 is a relatively weak motor protein.

There are at least six HCV genotypes that are prevalent in the world, infecting at least 2% of the total human population (1). These different viral genotypes result in diseases with various levels of severity, infectivity, response to treatment, and other attributes. Most of the previous structural and biochemical studies have focused on NS3 from genotype 1 (22–24), which is the most common form of infection in North America, although several studies have focused on NS3 from genotype 2a (25, 26). Among the strains of genotype 2a, the JFH-1 strain, isolated from a case of fulminant hepatitis, is a particularly efficient replicator (27, 28). Indeed, by using sequences from this strain, it was possible to create the first systems for studying the complete HCV replication cycle in cultured cells (29), which led to rapid advances in our understanding of the virus and the development of highly effective antiviral drugs.

Remarkably, despite the robust properties of the JFH-1 strain and its clear medical importance, mechanistic studies on the replicative proteins from genotype 2a strains have been lacking. Structural and biochemical studies have shown that the JFH-1 NS5B polymerase has distinctive features that promote robust replication (30), and NS3 contributes to replication in cell culture (27), but there has been scant attention to the enzymology of the JFH-1 NS3 helicase (31). We therefore wondered if the JFH-1 NS3 helicase is different from the well-studied NS3 from genotypes 1a and 1b and whether it might have enzymatic attributes that facilitate the unusually robust replication of JFH-1. JFH-1 NS3 shares 79 to 81% sequence identity with NS3 from the genotype 1 strains, suggesting that there is sufficient variation to support substantive differences in their function. To evaluate the proficiency of JFH-1 NS3 as an RNA helicase, we examined its ability to bind and unwind RNA substrates of various composition. In addition, we solved the crystal structure of JFH-1 NS3 at high resolution, enabling us to compare it with NS3 variants from genotype 1b. We observe that JFH-1 NS3 is a strikingly robust helicase and that it contains unique structural features that facilitate productive interactions with RNA.

## RESULTS AND DISCUSSION

### Design of RNA-unwinding substrates.

The activity of SF2 helicases is typically monitored on “tailed” duplex substrates that contain a duplex region flanked by a single-stranded nucleic acid (overhang) (7, 32, 33). The degree of functional oligomerization is typically reflected by the length of the 3′ overhang that is required for loading of helicase molecules at the unwinding initiation site (15, 17). In order to compare the behaviors of NS3 from two different genotypes (genotype 2a strain JFH1 versus genotype 1b strain N [34]), we designed a set of substrates that varied in duplex and overhang length and tested their relative RNA binding affinities and unwinding activities.

### Strong binding affinity and rapid association kinetics of JFH-1 NS3.

**FIG 1:**
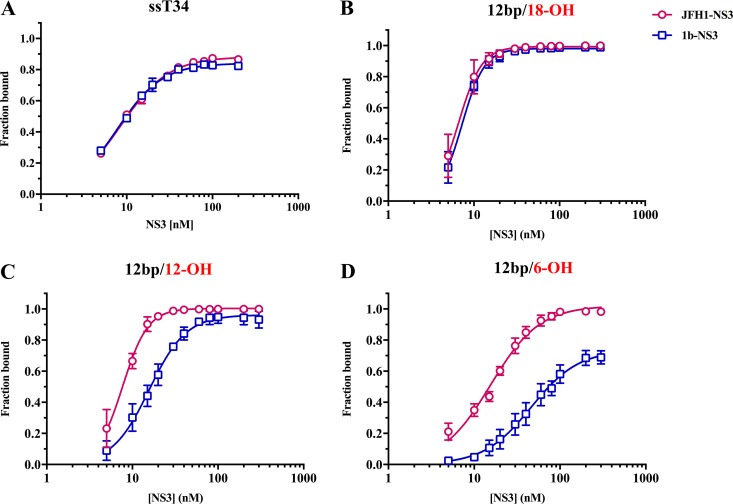
RNA binding affinities of JFH1-NS3 and gt1b-NS3 to RNAs with various overhang lengths. JFH1-NS3 and gt1b-NS3 variants are indicated with red circles and blue squares, respectively. RNA affinity was measured by filter binding, as described in Materials and Methods. (A) Affinity of JFH-1 NS3-4A (8.36 ± 0.29 nM) and gt1b NS3-4A (7.56 ± 0.33 nM) for single-stranded RNA (ssT34). (B) Affinity of JFH1-NS3 (6.56 ± 0.23 nM) and gt1b-NS3 (7.25 ± 0.17 nM) for a 12-base-pair duplex RNA with an 18-nt single-stranded 3′ overhang. (C) Affinity of JFH1-NS3 (7.69 ± 0.21 nM) and gt1b-NS3 (15.7 ± 0.61 nM) for a 12-base-pair duplex RNA with a 12-nt 3′ overhang. (D) Affinity of JFH1-NS3 (14.9 ± 0.52 nM) and gt1b-NS3 (60.4 ± 4.38 nM) for a 12-bp duplex RNA with a 6-nt 3′ overhang. The experiments were performed in triplicate, and error bars represent standard errors of the mean.

To evaluate the relative affinity of JFH-1 NS3 for RNA and to compare it with affinity of a well-studied genotype 1b strain N NS3 (here referred to as gt1b NS3), we first performed equilibrium filter binding experiments (10, 35) by using RNA substrates with various 3′-overhang lengths. We first examined the affinity of the JFH-1 NS3 and gt1b NS3 variants for a 34-nt single-strand RNA (ssT34), and observed similar *K_D_* (equilibrium dissociation constant) values of 7 to 8 nM in both cases (Fig. 1A), as reported previously for gt1b NS3 (10). We then examined NS3 binding to a set of 3′-overhang duplex substrates (described in Tables 1 and 2). For substrates containing a long, single-stranded 3′ overhang (as in the 12bp/18-OH substrate), both genotypes bound with similar affinity (Fig. 1B).

**TABLE 1:** RNA oligonucleotide sequences used in this study

Oligonucleotide no.	Sequence	Length (nt)
1	5′-GCC UCG CUG CCG-3′	12
2	5′-GCC UCG CUG CCG UCG CCA-3′	18
3	5′-CGG CAG CGA GGC AGA GGA GCA GAG GGA GCA-3′	30
4	5′-CGG CAG CGA GGC AGA GGA GCA GCA-3′	24
5	5′-CGG CAG CGA GGC AGG ACA-3′	18
6	5′-CGG CAG CGA GGC-AGA-3′	15
7	5′-UGG CGA CGG CAG CGA GGC AGA GGA GCA GAG GGA GCA-3′	36
8	5′-CGG CAG CGA GGC GAG GGA AGA GGA GCA GCA-3′	30
9	5′-UCC CUC GCC UCG CUG CCG-3′	18
10	5′-UGG CGA CGG CAG CGA GGC AGG ACA-3′	24
11	5′-CUG UGG CAU GUC CUA GCG UCG UAU CGA UCU GGU C-3′	34

**TABLE 2:** Assembly of RNA duplexes that were used in different assays

Assay	RNA design	Oligonucleotide pair no.^*a*^
Binding	12bp/18-OH	1 + 3
	12bp/12-OH	1 + 4
	12bp/6-OH	1 + 5
Unwinding	12bp/6-OH	1 + 5
	12bp/3-OH	1 + 6
	18bp/18-OH	2 + 7
	18bp/12-OH	8 + 9
	18bp/6-OH	2 + 10

a The oligonucleotide numbers are indicated in Table 1.

However, NS3 showed very different profiles of binding to duplexes that are flanked by shorter overhangs. When the RNA overhang length is shortened to 12 nt (12bp/12-OH), JFH-1 NS3 maintains high affinity, but the affinity of gt1b NS3 decreases (Fig. 1C). This gap in relative affinity becomes even more pronounced when the RNA overhang length is reduced to 6 nt (12bp/6-OH). In this case, one observes a small reduction in affinity of JFH-1 NS3 but a dramatic drop in affinity of gt1b NS3, resulting in a 4-fold difference between the two NS3 variants (Fig. 1D). These data indicate that JFH-1 NS3 has strong affinity for RNA duplexes with long overhangs, which bind at least two NS3 molecules, as observed previously. However, unlike gt1b NS3, JFH-1 NS3 maintains strong affinity even on a 6-nt overhang that can accommodate only a single NS3 molecule. This suggests that JFH-1 NS3 is functional as a monomer in the absence of cooperative binding.

**FIG 2:**
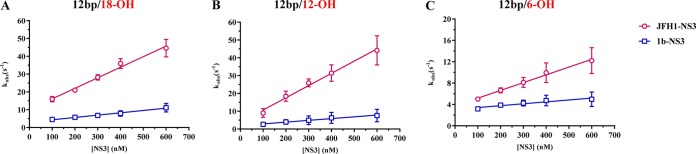
Association rate constants (*k*_on_) for JFH-1 NS3 and gt1b NS3 binding to RNAs with various overhang lengths. Rate constants were measured in a stopped-flow apparatus, as described in Materials and Methods. Data for JFH-1 NS3 and gt1b NS3 are indicated with red circles and blue squares, respectively. (A) The *k*_on_ values for JFH-1 NS3 and gt1b NS3 on a 12-bp duplex RNA with an 18-nt 3′ overhang are 59 ± 4.6 μM^−1^ s^−1^ and 13 ± 2.0 μM^−1^ s^−1^, respectively. (B) The *k*_on_ values for JFH-1 NS3 and gt1b NS3 on a 12-bp duplex RNA with a 12-nt 3′ overhang are 69 ± 6.4 μM^−1^ s^−1^ and 9.9 ± 3.3 μM^−1^ s^−1^, respectively. (C) The *k*_on_ values for JFH-1 NS3 and gt1b NS3 on a 12-bp duplex RNA with a 6-nt 3′ overhang are 15 ± 1.9 μM^−1^ s^−1^ and 3.5 ± 1.1 μM^−1^ s^−1^, respectively. The experiments were performed in triplicate, and error bars represent standard errors of the mean.

To obtain a quantitative understanding of NS3 association kinetics, we used stopped-flow fluorescence experiments to directly measure the association rate constant (*k*_on_) of the NS3 variants (36). Cy3-labeled substrate RNA (50 nM) was combined with NS3 protein (from 100 to 600 nM) under pseudo-first-order conditions ([NS3] > [RNA]), and the increase in fluorescent signal upon complex formation was monitored as a function of time after mixing. Values for *k*_obs_ were obtained at various protein concentrations, and these were plotted to obtain association rate constants as described in Materials and Methods. The resulting data show that JFH-NS3 binds all of the RNA substrates much more rapidly than gt1b NS3 (Fig. 2). RNA duplexes flanked by longer overhangs, of 12 and 18 nt, were bound particularly rapidly, although a rate reduction was observed for the 6-nt overhang, particularly in the case of gt1b NS3. These data indicate that JFH-1 NS3 binds RNA more rapidly than gt1b NS3, which correlates with the observed differences in equilibrium binding affinity.

### JFH-1 NS3 rapidly forms functional unwinding complexes with RNA.

A hallmark of gt1b NS3 helicase activity is the pronounced lag that is observed in the time required for the formation of functional unwinding complexes (14, 21). Given the higher binding rate and higher affinity of JFH-1 NS3 for RNA helicase substrates, it was of interest to determine whether the resulting complexes are functional as soon as they form and whether they form rapidly. Such behavior would contrast with that of gt1b NS3, which undergoes a slow, rate-limiting conformational change before unwinding. To address this issue, we directly monitored the rate constant for functional complex formation, as described previously (14, 21). In this assay, NS3 is incubated with RNA substrate for various amounts of time (1 to 60 min) prior to the addition of ATP and trap oligonucleotide (in excess, under single-cycle conditions), and then the reaction is stopped after a single, specific time interval (5 min).

**FIG 3:**
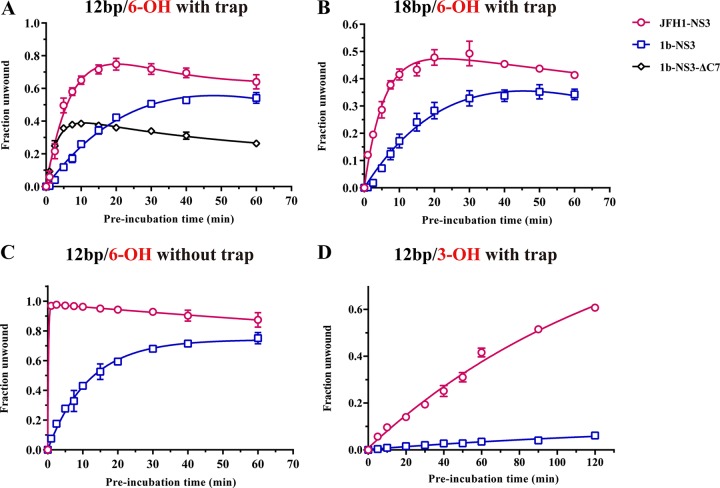
Rate constants for functional NS3-RNA complex formation. JFH1-NS3, gt1b-NS3, and gt1b-NS3-ΔC7 are indicated by red circles, blue squares, and black diamonds, respectively. (A) Functional complex formation between NS3 variants and a 12-bp duplex RNA with a 6-bp 3′ overhang, resulting in rate constants of *k*_1_ = 0.37 ± 0.08 min^−1^ and *k*_2_ = 0.61 ± 0.15 min^−1^ for JFH-1 NS3, *k*_1_ = 0.09 ± 0.01 min^−1^ and *k*_2_ = 0.36 ± 0.11 min^−1^ for gt1b-NS3, and *k*_2_ = 0.33 ± 0.03 min^−1^ for gt1b-NS3-ΔC7, as described in Materials and Methods. (B) Functional complex formation between NS3 variants and an 18-bp duplex RNA with a 6-bp 3′ overhang, resulting in rate constants of *k*_1_ = 0.23 ± 0.02 min^−1^ and *k*_2_ = 0.48 ± 0.11 min^−1^ for JFH1-NS3 and *k*_1_ = 0.11 ± 0.04 min^−1^ and *k*_2_ = 0.26 ± 0.06 min^−1^ for gt1b-NS3. (C) Rapid functional complex formation by JFH-1 NS3 in the absence of trap RNA (ssT34) on a 12-bp duplex RNA flanked by a 6-bp 3′ overhang. JFH1-NS3 completely unwinds the duplex extremely rapidly, with a rate constant that is higher than can be computed for this hand-mixed experiment (>1.4 min^−1^, which should be considered a lower bound on the rate constant), whereas gt1b-NS3 unwinds at a time scale similar to that for single-cycle conditions (0.09 ± 0.01 min^−1^). (D) Functional complex formation between NS3 variants and a 12-bp duplex RNA with a 3-nt 3′ overhang, resulting in rate constants of 300 ± 54 × 10^−4^ min^−1^ and 5.7 ± 0.49 × 10^−4^ min^−1^, for JFH-1 NS3 and gt1b NS3, respectively. Experiments were performed in triplicate, and error bars represent standard errors of the mean.

For both of the helicase substrates tested (12bp/6-OH and 18bp/6-OH), JFH-1 NS3 forms functional complexes 2- to 4-fold faster than gt1b NS3, and there is no lag in complex formation under single-cycle conditions (Fig. 3A and B), suggesting the lack of a rate-limiting conformational change. With the exception of a higher amplitude, which is consistent with an improved processivity for JFH-1 NS3, the kinetic behavior of JFH-1 NS3 is similar to that of the ΔC7 variant of gt1b NS3, which has an open conformation that is thought to stimulate productive binding (21) (Fig. 3A).

When trap is not added with the ATP (multiple-cycle conditions), differences between the NS3 variants become particularly striking. As observed previously on DNA duplexes (15), the unwinding behavior of gt1b NS3 looks similar in the presence and absence of trap, suggesting that dissociated gt1b NS3 does not readily rebind. However, when trap RNA is omitted from unwinding reactions of JFH-1 NS3 and unbound helicase molecules are free to participate in the subsequent reaction, the duplex is unwound completely and instantaneously, with a rate constant that is faster than can be measured in the design of this experiment (Fig. 3C). This type of behavior has never been observed before and may be linked to the rapid on-rate of JFH-1 NS3 molecules, which may be able to associate with tiny single-stranded overhangs that are present on the preincubated complex (see below). These data indicate that JFH-1 NS3 can initiate (and reinitiate) unwinding without any preincubation; in other words, unwinding by JFH-1 NS3 takes place immediately. This implies that JFH-1 NS3 can act on a broader selection of targets, including RNA duplexes that are flanked by only a few nucleotides.

### JFH-1 NS3 unwinds RNA duplexes with tiny 3′ overhangs.

Given the above observations and the fact that JFH-1 NS3 interacts avidly and productively with overhangs as short as 6 nt, we wondered if helicase activity might be maintained with even shorter overhangs. To explore this question, we conducted unwinding time courses by using a short duplex flanked by a 3′ overhang of three nucleotides (12bp/3-OH) (Fig. 3D). An additional motivation for examining shorter overhangs is that the 3′ untranslated region (UTR) of the HCV genome is highly structured (37, 38), but none of the many stable stem-loops in this region are flanked by the long RNA overhangs that are typical for the artificial substrates used in previous NS3 studies. Indeed, a very short overhang (1 to 3 nt) is likely to provide a more relevant physiological substrate for NS3, assuming that the helicase unwinds the viral genome.

In single-turnover unwinding studies, one observes that a significant amount of the 12bp/3-OH substrate is unwound by JFH-1 NS3, although no activity is observed for the gt1b NS3 helicase. In the first 10 min of reaction, approximately 10% of the RNA substrate was unwound by JFH-1 NS3 (Fig. 3D). When the reaction time is extended to 2 h, more than 60% of the 12bp/3-OH duplex is unwound (Fig. 3D). While the rates and amplitudes of this reaction are significantly lower than those for other substrates with longer overhangs examined in this study, they demonstrate that NS3 can initiate and successfully unwind RNA duplexes that are flanked by overhangs that have never supported activity by other SF2 helicases, including gt1b NS3. This unprecedented level of activity on such a short overhang suggests that, like certain SF1 helicases, the JFH-1 NS3 helicase monomer melts the first several base pairs adjacent to the 3′ overhang (39), enabling it to establish a firm grip on the loading strand and prime the unwinding reaction prior to addition of ATP.

### Relative rate constants and amplitudes for unwinding by JFH-1 NS3.

**FIG 4:**
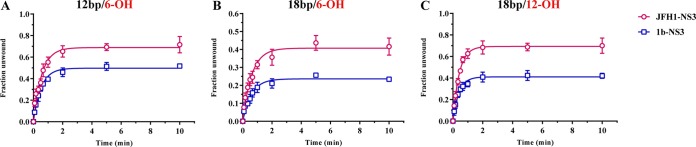
Single-cycle RNA unwinding efficiencies of JFH-1 NS3 and gt1b NS3 (A) The rate constants (*k*_unw_ values) and amplitudes (in parentheses) for unwinding a 12-bp RNA duplex with a 6-nt 3′ overhang are 1.8 ± 0.15 min^−1^ (0.69 ± 0.02) and 1.9 ± 0.11 min^−1^ (0.50 ± 0.01) for JFH-1 NS3 and gt1b NS3, respectively. (B) The rate constants and amplitudes for unwinding an 18-bp duplex RNA with a 6-nt 3′ overhang are 1.6 ± 0.17 min^−1^ (0.41 ± 0.01) and 1.7 ± 0.18 min^−1^ (0.24 ± 0.01) for JFH-1 NS3 and gt1b NS3, respectively. (C) The rate constants and amplitudes for unwinding an 18-mer duplex RNA with a 12-nt 3′ overhang are 2.5 ± 0.14 min^−1^ (0.69 ± 0.01) and 2.5 ± 0.22 min^−1^ (0.41 ± 0.01) for JFH-1 NS3 and gt1b NS3, respectively. Experiments were performed in triplicate, and error bars represent standard errors of the mean.

In order to compare the unwinding activities of JFH-1 NS3 and gt1b NS3 on duplexes that are recognized by both enzymes, we monitored the single-cycle unwinding rate constants and amplitudes on a minimal set of three duplexes that vary in overhang and duplex length (12bp/6-OH, 18bp/6-OH, and 18bp/12-OH) (Fig. 4). Under these conditions, in which RNA and enzyme are preincubated before the addition of ATP and trap RNA, we observe that the unwinding rate constants (*k*_unw_) for the two NS3 variants are almost identical. This suggests that once the helicase initiates and begins to unwind, the actual enzymatic activities of these enzymes are the same. What appears to differentiate NS3 of the two genotypes is the affinity for RNA and the ability of JFH-1 NS3 to function as a monomer.

Consistent with an overall enhancement in RNA affinity observed in the previous experiments, the JFH-1 NS3 variant shows a higher unwinding amplitude with all three RNA duplexes tested (Fig. 4), which is a hallmark of enhanced processivity. Under single-cycle conditions, a high amplitude indicates that the JFH-1 NS3 helicase is less likely to fall off the RNA tracking strand than the gt1b NS3 variant, consistent with its higher overall affinity for single-stranded RNA.

### Crystal structure of JFH-1 NS3-4A.

To better understand the molecular basis for the unusual biomechanical behavior of JFH-1 NS3-4A, we obtained a high-resolution crystal structure of the protein. Until now, structures of NS3 from genotype 2 strains have been unavailable, which is remarkable given their medical importance and the fact that most HCV viral genetics and cell culture work is being done with gt2a strain JFH-1. All structural information on NS3 derives from variants of genotypes 1a and 1b, which share only ∼80% sequence identity with genotype 2a, raising the possibility of significant drift in structure and function. We were specifically interested in determining whether structural features of JFH-1 NS3-4A might explain its avidity for RNA and its ability to latch onto very short flanking overhangs.

**FIG 5:**
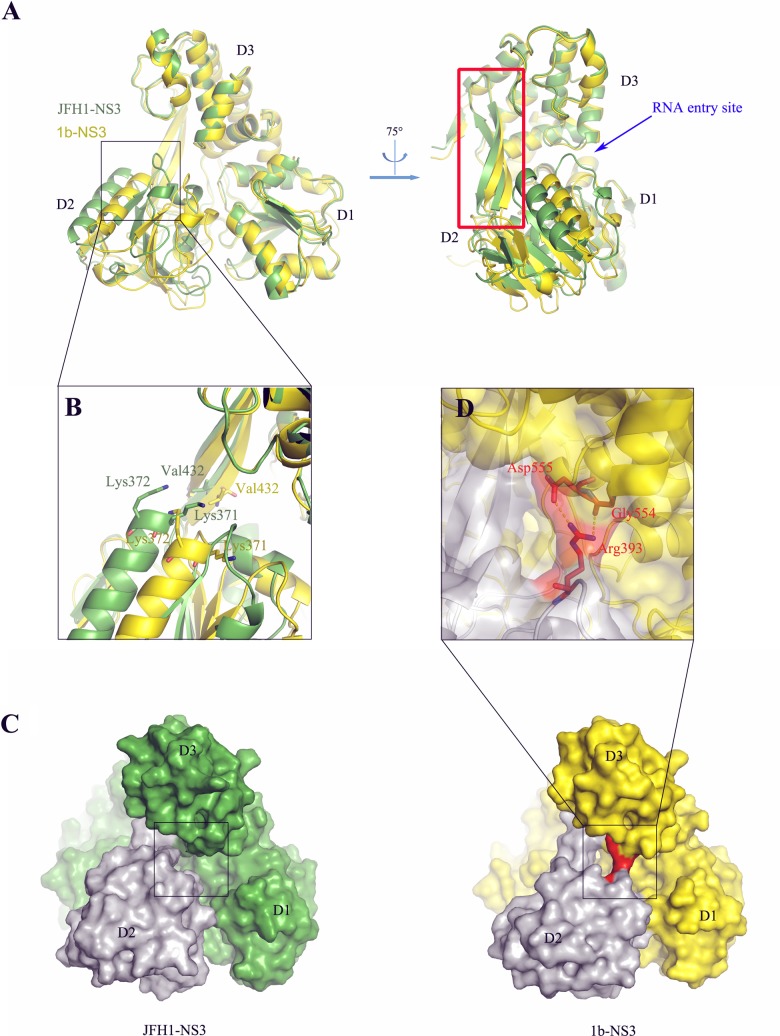
Structural comparison of JFH-1 NS3 and gt1b NS3 (PDB 3o8B). (A) Alignment of JFH-1 NS3 (green) and gt1b NS3 (yellow) in different views shows that the positional shift of D2 is caused by a 9.6° twist in the β14/15 hairpin (framed with a red rectangle). The RNA entry site is indicated with a blue arrow. (B) In the JFH-1 NS3 structure (green), the conserved RNA binding motifs IV (residues 365 to 372) and V (residues 411 to 419) shift outwards by about 6.8 Å and 4.2 Å, respectively, resulting in a longer RNA binding groove than in gt1b NS3 (yellow). (C) Comparison of the surfaces of JFH-1 NS3 (left panel) and gt1b NS3 (right panel). D2 is shown in gray for both variants. The gt1b NS3 variant contains an H-bond network (shown in red) that is absent in JFH-1 NS3, and the corresponding parts in both structures are indicated with a black square. (D) The H-bond network between D3 and D2 of gt1b NS3; a side chain atom of Asp555 and a main chain atom of Gly554 interact with side chain atoms of Arg393.

To carry out the structural studies, we used a conventional “single-chain” construct design in which JFH-1 NS4A amino acids 21 to 34 were fused via a short GSGS linker to the N terminus of JFH-NS3 (see Materials and Methods), yielding a full-length NS3 protein in which the β-sheet of the protease domain is stabilized and activated by intercalation of the NS4A fragment (22, 40). The recombinant construct is readily overexpressed in Escherichia coli, and the resulting fusion protein was isolated in high yield as a well-behaved monomer in solution, as indicated by size exclusion chromatography. The protein was crystallized by using the vapor diffusion method from a buffer containing 0.1 M Bis-Tris (pH 8.5), 20% polyethylene glycol (PEG) 3350, and 0.2 M sodium bromide, with a buffer containing an additional 25% glycerol as a cryoprotectant, which differs from conditions used to successfully crystallize Con-1 genotype 1b NS3 in terms of pH and salt type (80 mM Bis-Tris [pH 6.5], 0.16 M Li_2_SO_4_) (22). The resulting crystals diffracted to 2.23 Å, in space group P2_1_ 2_1_ 2_1_, and there is one molecule in the asymmetric unit (ASU). The ASU and the crystal contacts themselves differ from those observed in crystals of 1b NS3-4A (22). The JFH-1 NS3-4A structure was solved by molecular replacement using the apo structure of gt1b NS3-4A (Protein Data Bank [PDB] code 3o8B) as a search model (Fig. 5; Table 3).

**TABLE 3:** Data collection and refinement statistics

Parameter	Value^*a*^
Data collection	
Space group	P2_1_ 2_1_ 2_1_
Cell dimensions	
*a*, *b*, *c* (Å)	72.31, 89.42, 99.53
α, β, γ (°)	90, 90, 90
Resolution (Å)	58.3–2.23 (2.31–2.23)
*R*_sym_ or *R*_merge_	0.195 (2.241)
*R*_pim_	0.069 (0.791)
CC(1/2)	0.92 (0.88)
*I/σI*	10.8 (1.1)
Completeness (%)	100.0 (99.9)
Redundancy	8.8 (7.3)
Refinement	
Resolution (Å)	2.23
No. of reflections	31,814
*R*_work_/*R*_free_	0.212/0.238
No. of atoms	4,830
B factors (Å^2^)	48.59
Macromolecules	48.57
Ligands	41.35
Solvent	51.27
Root mean square deviations	
Bond lengths (Å)	0.026
Bond angles (°)	0.790
Ramachandran plot (%)	
Residues in most favored regions	96.0
Residues in allowed regions	4.0
Residues in disallowed regions	0.00

a Values in parentheses are for the highest-resolution shell.

### Conformational change is caused by a twist in the β14/β15 hairpin.

**FIG 6:**
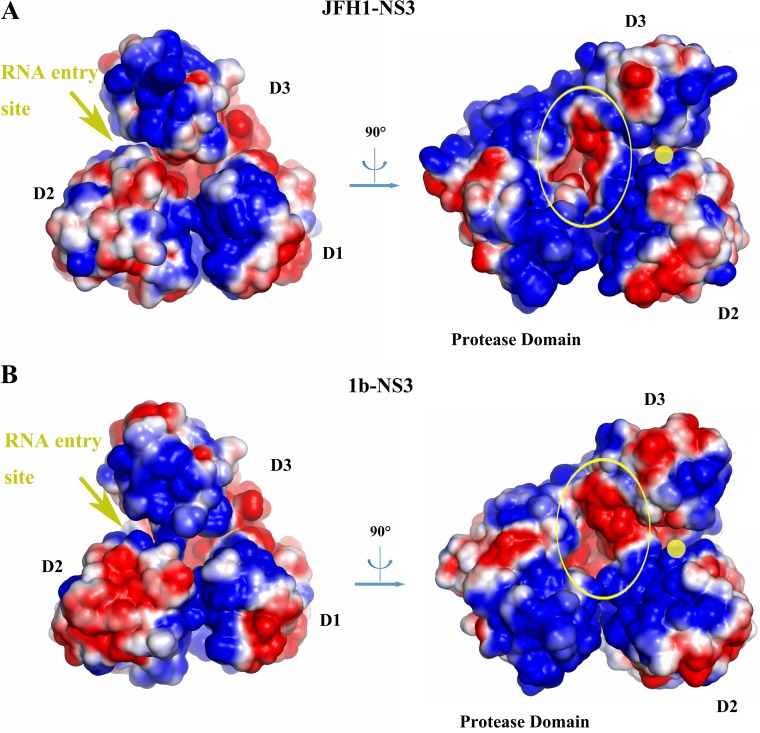
Comparison of the surface electrostatic potentials of JFH-1 NS3 (A) and gt1b NS3 (PDB 3o8B) (B). Left panels, RNA entry sites are indicated with yellow arrows. The surface of D2 has a smaller negatively charged patch adjacent to the RNA entry site in JFH-1 NS3 than in gt1b NS3, due to the twist of hairpin β14/15. Right panels, RNA entry sites are indicated with yellow dots. Both JFH-1 and gt1b NS3 contain a negatively charged pocket (yellow ellipse) near the RNA entry site. However, the rotation of D2 in JFH-1 NS3 causes this patch to be buried, resulting in a smaller presentation of negative charge. This decrease in surface-exposed negative charge may facilitate RNA attraction and better define RNA orientation upon binding to the protein.

The overall JFH-1 NS3-4A structure is composed of four globular domains arranged in a tetrahedral fashion, as observed previously for gt1b NS3-4A (22–24). These domains include an ATPase core (comprised of D1 and D2), which is capped by a third domain that completes the RNA binding cleft (D3). The protease domain, in complex with the NS4A peptide, is packed along the back of these three domains (Fig. 6A). When JFH-1 NS3-4A is superimposed on the structure of gt1b NS3-4A (PDB 3o8B) (22), one observes that D1, D3, and the protease domain are almost identical in conformation and positioning within both proteins (Fig. 5A). However, there is a pronounced shift in the position of D2, which is caused by a 9.6° twist in the conserved β14/15 hairpin (amino acids 430 to 452) (Fig. 5A). This hairpin has been shown to play a key role in RNA unwinding and ATP hydrolysis by SF2 helicase enzymes (41). The twist in the β hairpin propagates into a displacement of D2, altering its position by about 8.7 Å relative to the location of D2 in gt1b structures.

### JFH-1 NS3 has structural and electrostatic features conducive to RNA binding.

The conformational changes in D2 cause a remodeling of the electrostatic surface potential within the RNA binding site, particularly at the position in which RNA enters the helicase. For example, the negatively charged patch near the β14/15 hairpin is smaller in JFH-1 NS3 than in gt1b NS3 (Fig. 6). Most importantly, the displacement of D2 drives it closer to the protease domain (Fig. 6), creating a more narrow, concave area of positively charged residues between D2 and the protease domain. This positive cleft would be expected to improve early, productive contacts with RNA and to confine it in a specific orientation, potentially contributing to the faster and stronger RNA binding by JFH-1 NS3.

Another feature that is expected to alter encounters with RNA is that the β14/15 hairpin and Val432, which form part of the RNA “bookend” within the helicase cleft, move outward by 2.5 Å (Fig. 5B). Similarly, the conserved RNA binding motifs IV (residues 365 to 372) and V (residues 411 to 419) also shift outward by 6.8 Å and 4.2 Å, respectively (Fig. 5B). Together, these structural differences expand the length of the RNA binding tunnel and would enable JFH-1 NS3 to accommodate one more nucleotide than gt1b NS3, potentially explaining the higher RNA affinity and processivity of the JFH1 variant. Consistent with the distinctive conformation of this region, many amino acids that distinguish JFH-1 NS3 from gt1b lie predominantly within D2 and the β14/15 hairpin (see Table S1 in the supplemental material).

### JFH-1 NS3 lacks an obstruction in the RNA binding tunnel.

In addition to an altered spatial and electrostatic environment in the RNA binding cleft, the entrance to the RNA binding tunnel is markedly different in the two proteins. In structures of apo-gt1b NS3-4A, the entrance tunnel is obstructed by a network of interactions between conserved amino acids Arg393 of D2 and Gly554 and Asp555 of D3 (Fig. 5C, right panel, and D), resulting in a steric block that would restrict entry into the RNA binding cleft (22). This H-bond network disappears upon complexation of gt1b NS3-4A with RNA (PDB 3O8R), suggesting that it is poised to regulate the early stages of RNA binding and that gt1b NS3-4A must undergo conformational changes to productively interact with RNA ligands. In contrast, the RNA binding tunnel in apo-JFH-1 NS3-4A is completely open, resulting in unobstructed access to the RNA binding cleft (Fig. 5C, left panel). The shifted position of D2 prevents the formation of H bonds between Arg393, Gly554, and Glu555 in JFH-1 NS3-4A and frees the cleft to form a known set of interactions with the backbone of single-stranded RNA (23, 41). This unobstructed RNA binding site within apo-JFH-1 NS3-4A is expected to facilitate threading of a single-stranded RNA into the helicase core, and it is consistent with the rapid RNA binding, high affinity, and short overhang tolerance observed for JFH-1 NS3.

**FIG 7:**
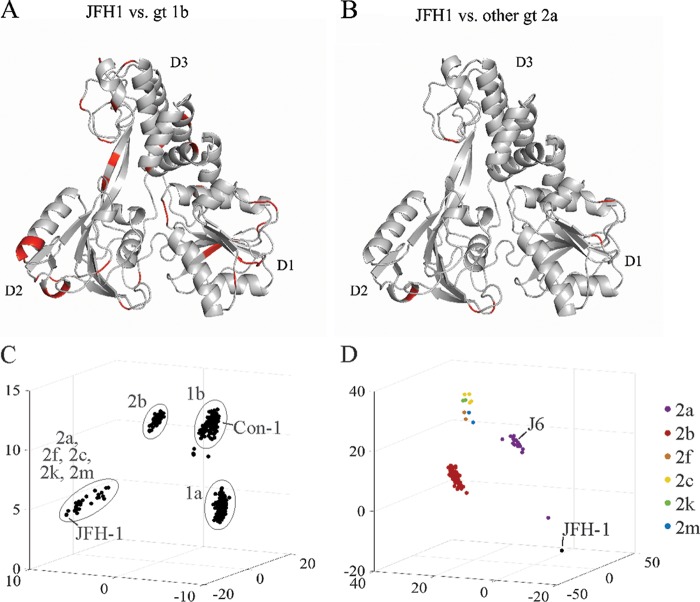
Comparison of the NS3 sequences from genotype 1 and 2 HCV strains. (A) Amino acid variations between JFH-1 and gt1b NS3-helicase domains are mapped on the JFH-1 NS3 structure (shown in red). (B) Amino acid variations between JFH-1 and other gt2a NS3-helicase domains are mapped on the JFH-1 NS3 structure (shown in red). The minor differences, such as I/L/V, S/T, or G/A substitutions, are not mapped in panel A or B. The statistics of the variations in both panels A and B are shown in Table S1 in the supplemental material. (C) NS3 sequences (801) from HCV genotypes 1 and 2 were analyzed by principal-component analysis, with the top three components graphed on *x*, *y*, and *z* axes. (D) NS3 sequences (123) from HCV genotype 2 were analyzed by principal-component analysis as for panel C.

It is important to highlight that it is the conformational shift of D2 which results in opening of the RNA binding cleft of JFH-1 compared to gt1b NS3 and not differences in the amino acid composition of the cleft itself. Indeed, the key residues at the entrance of this cleft are similar in the two proteins (having only a minor change of Asp555 in gt1b NS3 compared to Glu555 in JFH1-NS3). However, the exact identity of residues responsible for the conformational shift of D2 are not immediately obvious, as most of the variations among gt1 and gt2 strains are located at the periphery of NS3 in outward-facing loops or other regions and do not contribute in an obvious way to conformational differences between apo-JFH-1-NS3 and apo-1b-NS3 (Fig. 7; Table S1). A complex structure of JFH-1-NS3/RNA may be needed to shed light on the mechanism of enhanced JFH-1-NS3 activity.

### Phylogenetic relationships between JFH-1 and genotypes 1 and 2.

Having uncovered dramatic differences between the activities of gt1b and JFH-1 NS3, we sought to explore the evolutionary relatedness of NS3 from gt1 and gt2 sequences. To assess the variability of NS3 sequence, we performed principal-component analysis (PCA) of NS3 sequence alignments from these genotypes. As expected, subtypes segregated together, indicating increased variability between subtypes relative to intrasubtype variability (Fig. 7C). In this analysis, Con-1 NS3 falls within the grouping for gt1b, while JFH-1 falls at the periphery of the gt2a grouping. This led us to analyze how NS3 compares to the remainder of gt2. From a phylogenetic tree generated from gt2 NS3 alignments, we observed that the NS3 sequences from JFH-1 and a few highly similar strains segregate into a distinct group that is most closely related to the remainder of gt2a (see Fig. S1 in the supplemental material). However, PCA shows that while genotypes 2a, 2b, and 2c/f/k/m each segregate into individual clusters, JFH-1-like sequences form a separate cluster apart from other gt2a sequences (Fig. 7D). Notably, the commonly used J6 strain (42) segregates within gt2a. We conclude that although JFH-1 has traditionally been assigned to gt2a based on current classification criteria (43), it is not completely representative of this subgroup, and differences in NS3 might underlie the exceptional replication of JFH-1 in cell culture.

### Concluding remarks.

Although it has long been known that certain viral strains can replicate with greater efficiency, the molecular basis for this behavior has been unclear. Understanding the mechanisms underlying enhanced viral fitness is important because it can explain sudden outbreaks of viral disease and can highlight important therapeutic targets. Here we show that the NS3 helicase from the HCV JFH-1 strain contains sequence changes that enable it to bind rapidly and productively to a diversity of RNA unwinding targets, thereby transforming a relatively poor multimeric helicase (as in the case of NS3 from genotype 1b) to an unusually powerful monomeric helicase with enhanced ability to unwind the highly structured RNA viral genome (44). The molecular mechanism of enhanced RNA binding and unwinding is explained by the high-resolution crystal structure of JFH-1 NS3, which reveals a novel conformation in which the RNA binding cleft is open, unobstructed, and lined with a patch of positively charged residues, thereby enabling NS3-JFH-1 to load rapidly on RNA target sites within the HCV genome. Analysis of other genotype 2 NS3 variants will be interesting to determine if this enhanced activity is specific for closely related JFH-1 sequences or is a general property of genotype 2.

## MATERIALS AND METHODS

### RNA preparation.

RNA oligonucleotides were synthesized on an automated MerMade 6 synthesizer (BioAutomation, Irving, TX, USA) using standard phosphoramidite chemistry, deprotection, and purification procedures (45) (amidites were from Glen Research). Fluorophore-labeled “top-strand” RNAs were first synthesized by incorporating a C_3_ linker amino modifier amidite (Glen Research, catalog number 10-1913-90M) at the 5′ end. Amino-modified strands were then labeled by conjugation with Cy3 mono-*N*-hydroxysuccinimide (NHS) ester according to the manufacturer's instructions (GE Healthcare, Little Chalfont, United Kingdom). Cy3-labeled and unlabeled RNAs were separated on 20% denaturing polyacrylamide gels, eluted, and ethanol precipitated for storage. Radiolabeled RNA strands were 5′-end labeled with [γ-^32^P]ATP and T4 polynucleotide kinase and purified as described above. Unwinding substrates were prepared by annealing long “bottom-strand” oligonucleotides with short radiolabeled “top-strand” oligonucleotides in ME buffer (10 mM MOPS [morpholinepropanesulfonic acid] [pH 6.5], 50 mM NaCl, 1 mM EDTA), purifying on a native polyacrylamide gel and eluting as described previously (21). RNA concentrations were determined using a NanoDrop instrument (Thermo Scientific), using the corresponding calculated RNA extinction coefficients.

### Protein expression and purification.

Comparative RNA binding and unwinding studies were conducted with full-length NS3 proteins from genotype 1b (strain 1b-N; accession number AF139594) and genotype 2a (strain JFH1; accession number BAB32872) and with the NS3-1b-N-ΔC7 mutant (21), which were overexpressed and purified as previously described for genotype 1a NS3 (21). Crystallographic studies were conducted with a JFH-1 NS3-4A fusion protein that is analogous in design to gt1b NS3-4A fusion proteins described in the literature (46). All proteins were expressed by cloning the full-length sequence into the pET-SUMO vector, which introduces an N-terminal His_6_-SUMO tag that can later be cleaved by SUMO protease, leaving a native N terminus. Proteins were expressed in Rosetta II (DE3) cells (Novagen, Madison, WI, USA) by induction with 0.5 mM IPTG (isopropyl-β-d-thiogalactopyranoside) for 20 h at 16°C. Cells were then harvested by centrifugation at 6,000 rpm for 10 min at 4°C (with a Sorvall Lynx 6000 Superspeed centrifuge and a Fiberlite F9-6 ×1000 Lex carbon fiber rotor) and resuspended in lysis buffer (25 mM HEPES [pH 7.5], 300 mM NaCl, 5% glycerol, 5 mM β-mercaptoethanol [β-ME]) to a final volume of 150 ml per pellet (from 4 liters of culture). Cells were lysed in a microfluidizer at 5,000 lb/in^2^ for 2 cycles and then at 15,000 lb/in^2^ for 4 cycles. The lysate was then clarified by ultracentrifugation at 30,000 rpm for 30 min (with a Beckman Coulter Optima LE-80K ultracentrifuge and a type 45 Ti fixed-angle rotor). Soluble lysate was loaded onto 2.5 ml Ni-nitrilotriacetic acid (NTA) beads (Qiagen, Valencia, CA, USA) twice, washed with lysis buffer containing an additional 20 mM imidazole, and then eluted in a buffer of 25 mM HEPES (pH 7.5), 150 mM NaCl, 250 mM imidazole, 5% glycerol, and 5 mM β-mercaptoethanol. Eluted protein was combined with SUMO protease for 1 to 2 h at 4°C and diluted 4-fold with elution buffer (as described above) without imidazole to a final concentration of 50 mM imidazole. The cleaved protein was reloaded on freshly equilibrated Ni-NTA beads (1 ml) to remove the His_6_-SUMO tag. After flowing through the Ni-NTA column, the sample was loaded onto a HiTrap heparin HP column (GE Healthcare), washed with 25 mM HEPES (pH 7.5)–150 mM NaCl–5% glycerol–5 mM β-mercaptoethanol buffer, and eluted with the buffer containing 300 mM NaCl. The protein peak was collected and concentrated to ∼2 ml using a 30-kDa-molecular-mass-cutoff concentrator (Millipore, Billerica, MA, USA) and passed over a HiPrep 16/60 Superdex 200 column (GE Healthcare) in gel filtration buffer (25 mM HEPES [pH 7.5], 150 mM NaCl, 5% glycerol, 5 mM β-mercaptoethanol). Peak fractions were examined by SDS-PAGE and concentrated to 10 to 20 mg/ml. Concentrations were determined with a NanoDrop instrument (Thermo Scientific), and extinction coefficients were calculated using the ProtParam web server. Protein preparations were aliquoted, flash frozen using liquid nitrogen, and stored at −80°C.

### RNA binding studies.

NS3 affinity was monitored by filter binding, as described previously (10). Various concentrations of protein (from 5 to 300 nM, final concentration) were mixed with 0.5 nM (final concentration) of 5′-^32^P-end-labeled RNA substrates at 37°C in buffer (25 mM MOPS-NH_4_^+^ [pH 6.5], 3 mM MgCl_2_, 1% [vol/vol] glycerol, 2 mM dithiothreitol, 30 mM NaCl, 0.1% [vol/vol]Triton X-100) before loading onto a membrane sandwich. The membrane sandwich was composed of a top layer of nitrocellulose membrane (0.45 μm) (Pierce), a second layer of Nytran N nylon membrane (0.2 μm) (Whatman), and a bottom layer of filter paper (Whatman). A 50-μl aliquot of each incubation mixture was loaded on the dot blot apparatus while a vacuum was applied. The samples in each lane were washed three times using 100 μl of ice-cold assay buffer each time, and then the nitrocellulose and nylon membranes were quickly dried and the blots were exposed to a phosphorimager plate for several hours before being scanned with a Typhoon FLA 9500 biomolecular imager (GE). The percentage of the total RNA bound to protein was subsequently calculated. Experiments were performed in triplicate, and the data were fit to the Hill equation (Ys = [S]*n*/*K_d_* + [S]*n*) using GraphPad Prism.

To obtain RNA association rate constants (*k*_on_) for NS3 protein variants, stopped-flow fluorescence spectroscopy was employed. Stopped-flow experiments were performed in buffer (25 mM MOPS-NH_4_^+^ [pH 6.5], 3 mM MgCl_2_, 1% [vol/vol] glycerol, 2 mM dithiothreitol, 30 mM NaCl, 0.1% [vol/vol] Triton X-100) at 25°C using a Kintek Auto-SF 120 stopped-flow instrument (Kintek, Austin, TX, USA) equipped with a 150-W xenon arc lamp. For detection, the Cy3-labeled overhang RNA was excited at 515 nm and the fluorescence emission was monitored at ≥570 nm using a 570 band-pass filter (Newport Corporation, Irvine, CA, USA). Briefly, 500 μl of Cy3-labeled RNA (25 nM, final concentration) and 500 μl of NS3 at various concentrations (100, 200, 300, 400, and 600 nM, final concentration) were loaded into two injection syringes and rapidly mixed together for a specified period of time, after which data from 2,000 points were collected. The average fluorescence measurements (10 to 20 traces) for each condition were then used in data analysis. Experimental results were obtained from two independent experiments. Data were fit using nonlinear regression to single or double exponential equations in order to obtain *k*_obs_. The *k*_obs_ (high rate) values were then plotted versus NS3 protein concentration and fit to a line in order to obtain *k*_on_ values from the slope.

### RNA unwinding studies.

NS3 unwinding studies on the 3′-tailed duplexes were performed as previously described (14) with the following modifications. For unwinding reactions under single-cycle reaction conditions, a reaction mix containing 100 nM protein and 2 nM RNA duplex (final concentrations) was assembled in reaction buffer (25 mM MOPS-NH_4_ [pH 6.5], 3 mM MgCl_2_, 1% [vol/vol] glycerol, 2 mM dithiothreitol, 30 mM NaCl, 0.1% [vol/vol] Triton X-100) at room temperature for 30 min and then divided into 30-μl reaction aliquots. Each reaction mixture was incubated at 37°C for 1 h, and then 10 μl of an ATP-trap mixture (8 mM ATP and 4 μM protein trap in assay buffer) was added to initiate a single cycle of unwinding. The trap was a 34-nt, single-stranded RNA oligonucleotide described previously (21). For unwinding reactions conducted under multiple-cycle conditions, experiments were performed the same way but in the absence of trap RNA. Reactions were quenched at increasing time points (from 5 s to 10 min) by adding 10 μl of a 5× quench buffer (2.5× Tris-borate-EDTA [TBE], 100 mM EDTA, 1.25% SDS, 25% glycerol, and two loading dyes) to reaction aliquots, which were then immediately transferred to dry ice. Duplex and single-stranded RNAs were separated on semidenaturing polyacrylamide gels (described above), visualized with a Typhoon FLA 9500 biomolecular imager, and quantified with ImageQuant software (GE Healthcare). The fraction of unwound duplex at each time point was quantitated using the formula *A*_unw_ = *I*_ss_/(*I*_ss_ + *I*_ds_), where *I*_ss_ is the intensity of the displaced strand and *I*_ds_ is the intensity of the duplex substrate. The data were obtained from three independent experiments. Data were processed with GraphPad Prism and fit using a single exponential equation.

### Rate of functional complex formation.

Functional complex formation assays were performed as described in the literature (47), with the following modifications. Reaction mixtures were incubated at 37°C, and aliquots were taken at increasing times after mixing of RNA and protein (from 1 min to 60 min), prior to the addition of ATP and RNA trap, which were then incubated with each sample for 2 min. The resultant data were fit using equation 1 for genotype 1b-N and JFH-1 NS3 and to equation 2 for genotype 1b-N and ΔC7-NS3, as reported previously (21).

The data for gt1b-N/JFH-1 NS3 were modeled to scheme 1:

$${\text{NS}3}_{\text{c}}\overset{\text{RNA}}{⇄}{\text{NS}3}_{\text{c}}\cdot \text{RNA}\overset{k_{1}}{\to }{\text{NS}3}_{\text{e}}\cdot \text{RNA}\overset{k_{2}}{\to }[{\text{NS}3}_{\text{e}}\cdot \text{RNA}]*\overset{\text{fast}}{\to }\text{unwinding}$$

using equation 1:

(1) $$A_{\text{unw}}=A_{0}+\frac{A_{0}}{k_{2}-k_{1}}\left[k_{1}e^{-k_{2}t}-k_{2}e^{-k_{1}t}\right]$$

where *A*_unw_ is the unwinding amplitude at incubation time *t*, *A*_0_ is the apparent fraction of functional complex formed on the RNA substrate, *k*_1_ is the rate constant at which gt1b-N/JFH-1 NS3 undergoes an internal conformational change from a compact (NS3_c_) to an extended (NS3_e_) conformation, and *k*_2_ is the rate constant at which gt1b-N/JFH-1 NS3 forms a functional complex with RNA that leads to unwinding. The data for gt1b-NΔC7-NS3 were modeled to scheme 2:

$${NS3}_{c}\overset{\text{RNA}}{⇄}{NS3}_{c}\underset{decay}{\underset{↓k_{3}}{\cdot }}RNA\overset{k_{2}}{\to }[{NS3}_{e}\cdot RNA]*\overset{fast}{\to }\text{unwinding}$$

using equation 2:

(2) $$A_{\text{unw}}=A_{2}(1-e^{-k_{2}t})+A_{3}\left(1-e^{-k_{3}t}\right)$$

where *A*_unw_ is the unwinding amplitude at incubation time *t*, *A*_2_ is the amplitude of the fast exponential rise in activity with the rate constant *k*_2_, and *A*_3_ is the amplitude of the decay process with rate constant *k*_3_. Data were obtained from three independent experiments.

### Protein crystallization and structure determination.

JFH-1 NS3-4A protein crystallization screens were set up at 18°C by sitting-drop vapor diffusion. Protein concentrations used for crystallization ranged from 0.1 to 0.2 mM (7 to 14 mg/ml). Protein samples were mixed with crystallization reagents in equal volumes (0.2 μl plus 0.2 μl) using a Mosquito instrument (TTP Labtech). Visible needle crystals appeared in 3 days and grew to ∼0.1 mm in length within 2 weeks. Crystals grew from many different precipitants, such as PEG 3350 and ammonium acetate, but the best diffraction came from crystals grown in 0.1 M Bis-Tris (pH 8.5), 20% PEG 3350, and 0.2 M sodium bromide.

Diffraction data for JFH-1 NS3-4A was collected (at a wavelength of 0.97910 Å) at beamline 24ID-E (NE-CAT) at the Advanced Photon Source (APS) at Argonne National Labs. Data were collected at 100 K, using crystals cryoprotected by the addition of 25% glycerol in crystallization buffer. The data collection strategy and preliminary data processing were performed with the Rapid Automated Processing of Data (RAPD) software package (https://rapd.nec.aps.anl.gov/rapd/). The final indexing, integration, and scaling were performed with XDS. The structure was solved by molecular replacement with PHASER (48) using the genotype 1b (strain Con-1) structure (PDB 3o8B) as the search model. COOT (49) and PHENIX (50) were used for model fitting and refinement, respectively. Detailed statistics for data collection and refinement are shown in Table 3.

### Structural alignment.

Structural alignment and visualization were performed in PyMOL (51). As a starting point, the individual domains of JFH-1-NS3 and gt1b-NS3 (PDB 3o8B) were aligned, revealing that the structures of each individual domain are virtually identical except for the twist in the β14/15 hairpin of domain 2. Alignment of the entire structures was performed by superimposing domain 1 and domain 3, which results in the maximum match between these two structures.

### Phylogenetic analysis.

HCV genotype 1 and genotype 2 polyprotein sequences were downloaded from the NCBI Taxonomy Browser and aligned to the JFH-1 NS3 sequence using Clustal Omega (52). Alignments were viewed and edited in JalView (53) to crop sequences corresponding to NS3. Construction of phylogenetic trees and PCA was performed in JalView, and graphs were generated using MatLab.

## Supplementary Material

Supplemental material

## References

[1] Lindenbach BD, Thiel HJ, Rice CM 2007 Flaviviridae: the viruses and their replication, p 1101–1151. *In* Knipe DM, Howley PM (ed), Fields virology, 5th ed Lippincott Williams & Wilkins, Philadelphia, PA.

[2] Ma Y, Yates J, Liang Y, Lemon SM, Yi M 2008 NS3 helicase domains involved in infectious intracellular hepatitis C virus particle assembly. J Virol 82:7624–7639. doi:10.1128/JVI.00724-08. 18508894 PMC2493332

[3] Mukherjee S, Hanson AM, Shadrick WR, Ndjomou J, Sweeney NL, Hernandez JJ, Bartczak D, Li K, Frankowski KJ, Heck JA, Arnold LA, Schoenen FJ, Frick DN 2012 Identification and analysis of hepatitis C virus NS3 helicase inhibitors using nucleic acid binding assays. Nucleic Acids Res 40:8607–8621. doi:10.1093/nar/gks623. 22740655 PMC3458564

[4] McGivern DR, Masaki T, Lovell W, Hamlett C, Saalau-Bethell S, Graham B 2015 Protease inhibitors block multiple functions of the NS3/4A protease-helicase during the hepatitis C virus life cycle. J Virol 89:5362–5370. doi:10.1128/JVI.03188-14. 25740995 PMC4442512

[5] Saalau-Bethell SM, Woodhead AJ, Chessari G, Carr MG, Coyle J, Graham B, Hiscock SD, Murray CW, Pathuri P, Rich SJ, Richardson CJ, Williams PA, Jhoti H 2012 Discovery of an allosteric mechanism for the regulation of HCV NS3 protein function. Nat Chem Biol 8:920–925. doi:10.1038/nchembio.1081. 23023261 PMC3480716

[6] Mukherjee S, Weiner WS, Schroeder CE, Simpson DS, Hanson AM, Sweeney NL, Marvin RK, Ndjomou J, Kolli R, Isailovic D, Schoenen FJ, Frick DN 2014 Ebselen inhibits hepatitis C virus NS3 helicase binding to nucleic acid and prevents viral replication. ACS Chem Biol 9:2393–2403. doi:10.1021/cb500512z. 25126694 PMC4201343

[7] Pyle AM 2008 Translocation and unwinding mechanisms of RNA and DNA helicases. Annu Rev Biophys 37:317–336. doi:10.1146/annurev.biophys.37.032807.125908. 18573084

[8] Ding SC, Pyle AM 2012 Molecular mechanics of RNA translocases. Methods Enzymol 511:131–147. doi:10.1016/B978-0-12-396546-2.00006-1. 22713318 PMC4407658

[9] Levin MK, Gurjar M, Patel SS 2005 A Brownian motor mechanism of translocation and strand separation by hepatitis C virus helicase. Nat Struct Mol Biol 12:429–435. doi:10.1038/nsmb920. 15806107

[10] Beran RK, Serebrov V, Pyle AM 2007 The serine protease domain of hepatitis C viral NS3 activates RNA helicase activity by promoting the binding of RNA substrate. J Biol Chem 282:34913–34920. doi:10.1074/jbc.M707165200. 17921146

[11] Beran RK, Pyle AM 2008 Hepatitis C viral NS3-4A protease activity is enhanced by the NS3 helicase. J Biol Chem 283:29929–29937. doi:10.1074/jbc.M804065200. 18723512 PMC2573085

[12] Frick DN, Rypma RS, Lam AM, Gu B 2004 The nonstructural protein 3 protease/helicase requires an intact protease domain to unwind duplex RNA efficiently. J Biol Chem 279:1269–1280. doi:10.1074/jbc.M310630200. 14585830 PMC3571687

[13] Rajagopal V, Gurjar M, Levin MK, Patel SS 2010 The protease domain increases the translocation stepping efficiency of the hepatitis C virus NS3-4A helicase. J Biol Chem 285:17821–17832. doi:10.1074/jbc.M110.114785. 20363755 PMC2878546

[14] Pang PS, Jankowsky E, Planet PJ, Pyle AM 2002 The hepatitis C viral NS3 protein is a processive DNA helicase with cofactor enhanced RNA unwinding. EMBO J 21:1168–1176. doi:10.1093/emboj/21.5.1168. 11867545 PMC125889

[15] Tackett AJ, Chen Y, Cameron CE, Raney KD 2005 Multiple full-length NS3 molecules are required for optimal unwinding of oligonucleotide DNA in vitro. J Biol Chem 280:10797–10806. doi:10.1074/jbc.M407971200. 15634684

[16] Dumont S, Cheng W, Serebrov V, Beran RK, Tinoco I Jr, Pyle AM, Bustamante C 2006 RNA translocation and unwinding mechanism of HCV NS3 helicase and its coordination by ATP. Nature 439:105–108. doi:10.1038/nature04331. 16397502 PMC1560093

[17] Serebrov V, Beran RK, Pyle AM 2009 Establishing a mechanistic basis for the large kinetic steps of the NS3 helicase. J Biol Chem 284:2512–2521. doi:10.1074/jbc.M805460200. 19010782 PMC2629105

[18] Serebrov V, Pyle AM 2004 Periodic cycles of RNA unwinding and pausing by hepatitis C virus NS3 helicase. Nature 430:476–480. doi:10.1038/nature02704. 15269774

[19] Levin MK, Patel SS 1999 The helicase from hepatitis C virus is active as an oligomer. J Biol Chem 274:31839–31846. doi:10.1074/jbc.274.45.31839. 10542208

[20] Levin MK, Wang YH, Patel SS 2004 The functional interaction of the hepatitis C virus helicase molecules is responsible for unwinding processivity. J Biol Chem 279:26005–26012. doi:10.1074/jbc.M403257200. 15087464

[21] Ding SC, Kohlway AS, Pyle AM 2011 Unmasking the active helicase conformation of nonstructural protein 3 from hepatitis C virus. J Virol 85:4343–4353. doi:10.1128/JVI.02130-10. 21325413 PMC3126282

[22] Appleby TC, Anderson R, Fedorova O, Pyle AM, Wang R, Liu X, Brendza KM, Somoza JR 2011 Visualizing ATP-dependent RNA translocation by the NS3 helicase from HCV. J Mol Biol 405:1139–1153. doi:10.1016/j.jmb.2010.11.034. 21145896 PMC3134248

[23] Kim JL, Morgenstern KA, Griffith JP, Dwyer MD, Thomson JA, Murcko MA, Lin C, Caron PR 1998 Hepatitis C virus NS3 RNA helicase domain with a bound oligonucleotide: the crystal structure provides insights into the mode of unwinding. Structure 6:89–100. doi:10.1016/S0969-2126(98)00010-0. 9493270

[24] Cho HS, Ha NC, Kang LW, Chung KM, Back SH, Jang SK, Oh BH 1998 Crystal structure of RNA helicase from genotype 1b hepatitis C virus. A feasible mechanism of unwinding duplex RNA. J Biol Chem 273:15045–15052. 9614113 10.1074/jbc.273.24.15045

[25] Lam AM, Keeney D, Eckert PQ, Frick DN 2003 Hepatitis C virus NS3 ATPases/helicases from different genotypes exhibit variations in enzymatic properties. J Virol 77:3950–3961. doi:10.1128/JVI.77.7.3950-3961.2003. 12634355 PMC150621

[26] Li YP, Ramirez S, Gottwein JM, Scheel TK, Mikkelsen L, Purcell RH, Bukh J 2012 Robust full-length hepatitis C virus genotype 2a and 2b infectious cultures using mutations identified by a systematic approach applicable to patient strains. Proc Natl Acad Sci U S A 109:E1101–E1110. doi:10.1073/pnas.1203829109. 22467829 PMC3344947

[27] Murayama A, Date T, Morikawa K, Akazawa D, Miyamoto M, Kaga M, Ishii K, Suzuki T, Kato T, Mizokami M, Wakita T 2007 The NS3 helicase and NS5B-to-3′X regions are important for efficient hepatitis C virus strain JFH-1 replication in Huh7 cells. J Virol 81:8030–8040. doi:10.1128/JVI.02088-06. 17522229 PMC1951293

[28] Binder M, Quinkert D, Bochkarova O, Klein R, Kezmic N, Bartenschlager R, Lohmann V 2007 Identification of determinants involved in initiation of hepatitis C virus RNA synthesis by using intergenotypic replicase chimeras. J Virol 81:5270–5283. doi:10.1128/JVI.00032-07. 17344294 PMC1900214

[29] Lindenbach BD, Evans MJ, Syder AJ, Wolk B, Tellinghuisen TL, Liu CC, Maruyama T, Hynes RO, Burton DR, McKeating JA, Rice CM 2005 Complete replication of hepatitis C virus in cell culture. Science 309:623–626. doi:10.1126/science.1114016. 15947137

[30] Schmitt M, Scrima N, Radujkovic D, Caillet-Saguy C, Simister PC, Friebe P, Wicht O, Klein R, Bartenschlager R, Lohmann V, Bressanelli S 2011 A comprehensive structure-function comparison of hepatitis C virus strain JFH1 and J6 polymerases reveals a key residue stimulating replication in cell culture across genotypes. J Virol 85:2565–2581. doi:10.1128/JVI.02177-10. 21209117 PMC3067924

[31] Appleby TC, Perry JK, Murakami E, Barauskas O, Feng J, Cho A, Fox D III, Wetmore DR, McGrath ME, Ray AS, Sofia MJ, Swaminathan S, Edwards TE 2015 Viral replication. Structural basis for RNA replication by the hepatitis C virus polymerase. Science 347:771–775. doi:10.1126/science.1259210. 25678663

[32] Jankowsky E, Gross CH, Shuman S, Pyle AM 2000 The DExH protein NPH-II is a processive and directional motor for unwinding RNA. Nature 403:447–451. doi:10.1038/35000239. 10667799

[33] Ali JA, Lohman TM 1997 Kinetic measurement of the step size of DNA unwinding by Escherichia coli UvrD helicase. Science 275:377–380. doi:10.1126/science.275.5298.377. 8994032

[34] Beard MR, Abell G, Honda M, Carroll A, Gartland M, Clarke B, Suzuki K, Lanford R, Sangar DV, Lemon SM 1999 An infectious molecular clone of a Japanese genotype 1b hepatitis C virus. Hepatology 30:316–324. doi:10.1002/hep.510300137. 10385673

[35] Wong I, Lohman TM 1993 A double-filter method for nitrocellulose-filter binding: application to protein-Nucleic acid interactions. Proc Natl Acad Sci U S A 90:5428–5432. doi:10.1073/pnas.90.12.5428. 8516284 PMC46733

[36] Kozlov AG, Lohman TM 2002 Stopped-flow studies of the kinetics of single-stranded DNA binding and wrapping around the Escherichia coli SSB tetramer. Biochemistry 41:6032–6044. doi:10.1021/bi020122z. 11993998

[37] Kolykhalov AA, Feinstone SM, Rice CM 1996 Identification of a highly conserved sequence element at the 3′ terminus of hepatitis C virus genome RNA. J Virol 70:3363–3371. 8648666 10.1128/jvi.70.6.3363-3371.1996 PMC190207

[38] Yi M, Lemon SM 2003 Structure-function analysis of the 3′ stem-loop of hepatitis C virus genomic RNA and its role in viral RNA replication. RNA 9:331–345. doi:10.1261/rna.2144203. 12592007 PMC1370400

[39] Singleton MR, Dillingham MS, Gaudier M, Kowalczykowski SC, Wigley DB 2004 Crystal structure of RecBCD enzyme reveals a machine for processing DNA breaks. Nature 432:187–193. doi:10.1038/nature02988. 15538360

[40] Laughrey ZR, Kiehna SE, Riemen AJ, Waters ML 2008 Carbohydrate-pi interactions: what are they worth? J Am Chem Soc 130:14625–14633. doi:10.1021/ja803960x. 18844354 PMC2649776

[41] Lam AM, Keeney D, Frick DN 2003 Two novel conserved motifs in the hepatitis C virus NS3 protein critical for helicase action. J Biol Chem 278:44514–44524. doi:10.1074/jbc.M306444200. 12944414 PMC3571693

[42] Okamoto H, Okada S, Sugiyama Y, Kurai K, Iizuka H, Machida A, Miyakawa Y, Mayumi M 1991 Nucleotide sequence of the genomic RNA of hepatitis C virus isolated from a human carrier: comparison with reported isolates for conserved and divergent regions. J Gen Virol 72:2697–2704. doi:10.1099/0022-1317-72-11-2697. 1658196

[43] Smith DB, Bukh J, Kuiken C, Muerhoff AS, Rice CM, Stapleton JT, Simmonds P 2014 Expanded classification of hepatitis C virus into 7 genotypes and 67 subtypes: updated criteria and genotype assignment web resource. Hepatology 59:318–327. doi:10.1002/hep.26744. 24115039 PMC4063340

[44] Pirakitikulr N, Kohlway A, Lindenbach BD, Pyle AM 2016 The coding region of the HCV genome contains a network of regulatory RNA structures. Mol Cell 62:111–120. doi:10.1016/j.molcel.2016.01.024. 26924328 PMC4826301

[45] Wincott F, DiRenzo A, Shaffer C, Grimm S, Tracz D, Workman C, Sweedler D, Gonzalez C, Scaringe S, Usman N 1995 Synthesis, deprotection, analysis and purification of RNA and ribozymes. Nucleic Acids Res 23:2677–2684. doi:10.1093/nar/23.14.2677. 7544462 PMC307092

[46] Yao N, Reichert P, Taremi SS, Prosise WW, Weber PC 1999 Molecular views of viral polyprotein processing revealed by the crystal structure of the hepatitis C virus bifunctional protease-helicase. Structure 7:1353–1363. doi:10.1016/S0969-2126(00)80025-8. 10574797

[47] Beran RKF, Bruno MM, Bowers HA, Jankowsky E, Pyle AM 2006 Robust translocation along a molecular monorail: the NS3 helicase from hepatitis C virus traverses unusually large disruptions in its track. J Mol Biol 358:974–982. doi:10.1016/j.jmb.2006.02.078. 16569413

[48] McCoy AJ, Grosse-Kunstleve RW, Adams PD, Winn MD, Storoni LC, Read RJ 2007 Phaser crystallographic software. J Appl Crystallogr 40:658–674. doi:10.1107/S0021889807021206. 19461840 PMC2483472

[49] Emsley P, Cowtan K 2004 Coot: model-building tools for molecular graphics. Acta Crystallogr D Biol Crystallogr 60:2126–2132. doi:10.1107/S0907444904019158. 15572765

[50] Adams PD, Afonine PV, Bunkoczi G, Chen VB, Davis IW, Echols N, Headd JJ, Hung LW, Kapral GJ, Grosse-Kunstleve RW, McCoy AJ, Moriarty NW, Oeffner R, Read RJ, Richardson DC, Richardson JS, Terwilliger TC, Zwart PH 2010 PHENIX: a comprehensive Python-based system for macromolecular structure solution. Acta Crystallogr D Biol Crystallogr 66:213–221. doi:10.1107/S0907444909052925. 20124702 PMC2815670

[51] DeLano WL 2002 The PyMOL molecular graphics system. DeLano Scientific, Palo Alto, CA.

[52] Sievers F, Higgins DG 2017 Clustal Omega for making accurate alignments of many protein sequences. Protein Sci doi:10.1002/pro.3290. PMC5734385 28884485

[53] Waterhouse AM, Procter JB, Martin DM, Clamp M, Barton GJ 2009 Jalview version 2—a multiple sequence alignment editor and analysis workbench. Bioinformatics 25:1189–1191. doi:10.1093/bioinformatics/btp033. 19151095 PMC2672624

