# Supplementary material for: NS3 from Hepatitis C Virus Strain JFH-1 Is an Unusually Robust Helicase That Is Primed To Bind and Unwind Viral RNA
Source: J Virol. 2017 Dec 14;92(1):e01253-17. doi: 10.1128/JVI.01253-17 (PMC5730761; doi:10.1128/JVI.01253-17)
Supplement: Supplemental material [file supp_92_1_e01253-17__index.html]

NS3 from Hepatitis C Virus Strain JFH-1 Is an Unusually Robust Helicase That Is Primed To Bind and Unwind Viral RNA — Supplemental material 

# NS3 from Hepatitis C Virus Strain JFH-1 Is an Unusually Robust Helicase That Is Primed To Bind and Unwind Viral RNA

## Supplemental material

- Supplemental file 1 -

  Table S1 (Protein sequence variations derived from alignment of JFH-1 and other gt2a, gt2, and gt1 NS3 molecules.)

  Fig. S1 (Phylogenetic tree of gt2 NS3 sequence alignments.)

  PDF, 854K
