# Supplementary material for: NS3 from Hepatitis C Virus Strain JFH-1 Is an Unusually Robust Helicase That Is Primed To Bind and Unwind Viral RNA
Source: J Virol. 2017 Dec 14;92(1):e01253-17. doi: 10.1128/JVI.01253-17 (PMC5730761; doi:10.1128/JVI.01253-17)

Supplementary Table 1

Protein sequence variations that are derived from the alignment among JFH-1 and other gt2a, gt2 and gt1 NS3 molecules. The minor differences, like I/L/V, S/T or G/A substitutions are not included in this table of statistics.

<table>
<tr><th>position/function</th><th>aa</th><th>JFH-1</th><th>other gt2a</th><th>other gt2</th><th>gt1</th></tr>
<tr><td rowspan="14">Protease domain</td><td>49</td><td>A</td><td>S</td><td>S</td><td></td></tr>
<tr><td>67</td><td>L</td><td>S</td><td>S/P</td><td>P</td></tr>
<tr><td>77</td><td colspan="3">S</td><td>N</td></tr>
<tr><td>78</td><td colspan="3">A</td><td>V</td></tr>
<tr><td>80</td><td colspan="3">G</td><td>Q</td></tr>
<tr><td>95</td><td colspan="3">E/D</td><td>T</td></tr>
<tr><td>98</td><td>K</td><td colspan="3">T</td></tr>
<tr><td>102</td><td colspan="3">V</td><td>S</td></tr>
<tr><td>110</td><td>N</td><td colspan="3">H</td></tr>
<tr><td>122</td><td colspan="2">K</td><td>R</td><td>S</td></tr>
<tr><td>125</td><td colspan="3">A</td><td>S</td></tr>
<tr><td>134</td><td colspan="3">T/S</td><td>Y</td></tr>
<tr><td>150</td><td>V</td><td colspan="3">A</td></tr>
<tr><td>177</td><td colspan="3">V/I</td><td>T</td></tr>
<tr><td rowspan="5">Domain 1</td><td>216</td><td colspan="2">V</td><td colspan="2">A</td></tr>
<tr><td>242</td><td colspan="2">L</td><td colspan="2">M</td></tr>
<tr><td>260</td><td>M</td><td colspan="3">T</td></tr>
<tr><td>281</td><td>S</td><td>A</td><td colspan="2">G</td></tr>
<tr><td>295</td><td colspan="3">V</td><td>T</td></tr>
<tr><td rowspan="4">Domain 2</td><td>331</td><td colspan="3">T</td><td>V</td></tr>
<tr><td>334</td><td>P</td><td colspan="2">P/S</td><td>P/S</td></tr>
<tr><td>358</td><td>C</td><td>N/Y/S/F</td><td>N/Y/C/N</td><td>T/A/V/N</td></tr>
<tr><td>380</td><td colspan="3">A</td><td>K</td></tr>
<tr><td rowspan="4">β14/15 hairpin</td><td>430</td><td colspan="3">V</td><td>T</td></tr>
<tr><td>431</td><td colspan="3">A</td><td>C</td></tr>
<tr><td>447</td><td colspan="3">T</td><td>E</td></tr>
<tr><td>449</td><td colspan="3">Q</td><td>I/T</td></tr>
<tr><td>Domain 2</td><td>470</td><td>Q</td><td colspan="2">L</td><td>M/G/R/A/P</td></tr>
<tr><td rowspan="7">Domain 3</td><td>489</td><td colspan="3">V</td><td>S</td></tr>
<tr><td>499</td><td colspan="3">A</td><td>C</td></tr>
<tr><td>517</td><td colspan="3">F</td><td>M/L</td></tr>
<tr><td>572</td><td colspan="3">K</td><td>Q</td></tr>
<tr><td>580</td><td>A</td><td colspan="2">V</td><td>Q</td></tr>
<tr><td>606</td><td colspan="3">T</td><td>Q</td></tr>
<tr><td>620</td><td colspan="3">A</td><td>M</td></tr>
<tr><td></td><td>630</td><td colspan="3">M</td><td>V</td></tr>
</table>

Supplementary Figure 1

Phylogenetic tree of gt2 NS3 sequence alignments.

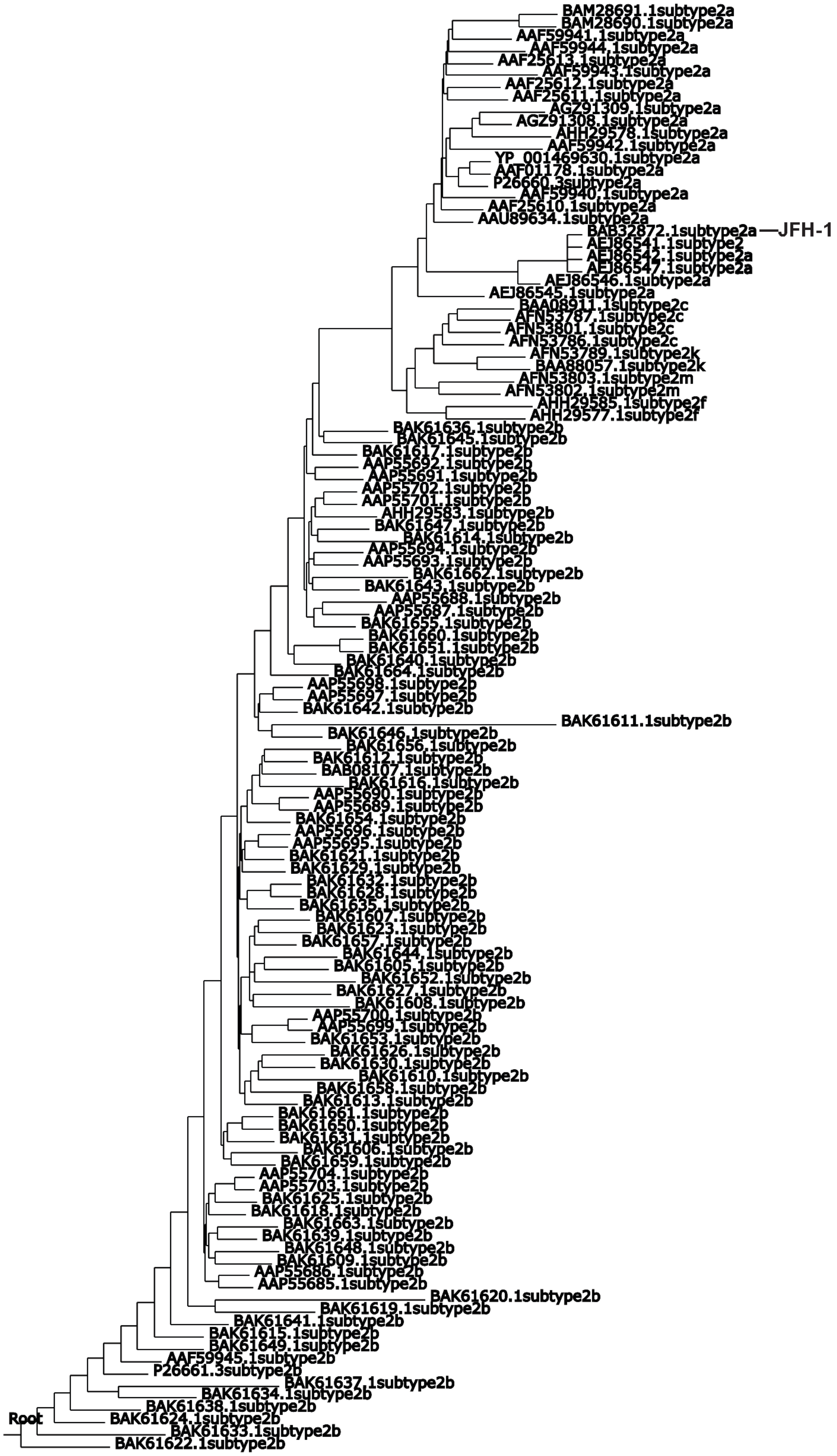

Supplement: Supplemental material [file JVI.01253-17_zjv001183205s1.pdf]
